# Effect of Interlayer on Flatness and Adhesion of Aerosol-Deposited Yttrium Oxide Coating

**DOI:** 10.3390/ma17143533

**Published:** 2024-07-17

**Authors:** Ki-Seong Lim, Tae-Soo Jang, Jae-hyeon Jeong, Sung-Hwan Hong, Joo Jin

**Affiliations:** 1 KoMiCo Co., Ltd., 8 Mosan-ro, Anseong-si 29271, Gyeonggi-do, Republic of Korea; taesoo.jang@komico.com (T.-S.J.); jaehyun.jung@komico.com (J.-h.J.); 2 Department of Nanotechnology and Advanced Materials Engineering, Sejong University, 209 Neungdong-ro, Gwangjin-gu, Seoul 05006, Republic of Korea; shhong@sejong.ac.kr; 3 MiCo Co., Ltd., 67 Dongtansandan 2-gil, Hwaseong-si 18487, Gyeonggi-do, Republic of Korea

**Keywords:** aerosol deposition, yttrium oxide, plasma-resistant coating, mechanical properties, adhesion

## Abstract

In this study, Y_2_O_3_ coating is used as an interlayer between Al_2_O_3_ substrate and a ceramic coating; this is in order to minimize the morphological distortion produced by a single deposition of the ceramic coating on the Al_2_O_3_ substrate, which is performed using the aerosol method. The interlayer coating, which comprises the Y_2_O_3_ phase, is deposited on the Al_2_O_3_ substrate using an e-beam evaporator. The crystal structure of the powder that was used to process the coating is identified as cubic Y_2_O_3_. In contrast, the crystal structure of the top-coating layer and interlayer indicates the presence of two kinds of Y_2_O_3_ phases, which possess cubic and monoclinic structures. The single Y_2_O_3_ coating without an interlayer exhibits microcracks around the interface between the coating and the substrate, which can be attributed to the stress that occurs during aerosol deposition. In contrast, no cracks are found in the aerosol-deposited Y_2_O_3_ coating and interlayer, which show a desirable microstructure. The single Y_2_O_3_ coating and the Y_2_O_3_ coating with an interlayer exhibit similar hardness and elastic modulus values. Nevertheless, the Y_2_O_3_ coating with an interlayer exhibits a higher level of adhesion than the single Y_2_O_3_ coating, with a value of 14.8 N compared to 10.2 N.

## 1. Introduction

During the manufacture of semiconductors and displays, plasma is widely utilized in cleaning and dry etching processes. It affects both the etching target and the internal parts of the manufacturing equipment employed in plasma etching. In particular, reactive gases containing fluorine generate many byproducts and contaminated particles during the dry etching process, which can cause critical problems and decrease the yield of semiconductor devices [1,2,3,4,5,6]. To prevent the corrosion of parts, plasma-resistant materials such as alumina (Al_2_O_3_), quartz (SiO_2_), and yttria (Y_2_O_3_) have been used to create the inner components of plasma etching equipment, such as the inner wall, window, shower head, and focus ring [5,6,7,8,9,10]. In particular, yttria has exhibited promise as an excellent plasma-resistant material compared with alumina and quartz in the plasma etching process when using reactive gas containing fluorine. However, the use of yttria for the inner parts of the chamber itself is too expensive, so it is mainly used to coat their surfaces.

The plasma-resistant coating on the inner parts of the chamber have been processed via anodization, atmospheric plasma spray (APS), suspension plasma spray (SPS), and aerosol deposition (AD) [11,12,13,14]. Among them, AD coatings have been reported to exhibit a denser structure and higher plasma resistance than thermal spray coatings. Moreover, AD coating is known to represent an innovative method that can be utilized in the fabrication of high-density ceramic coatings at room temperature under a vacuum atmosphere [15,16,17,18,19]. In this method, submicron-size ceramic particles are accelerated by carrier gas through a nozzle and sprayed onto a substrate with a velocity totaling a few hundred meters per second [5]. At the moment of collision, the kinetic energy of the ceramic particle is instantly transformed into bonding energy between the ceramic particle and the substrate, as well as between the ceramic particles without an additional heating system [20]. The main deposition mechanism is a hammering effect, which affects the formation of a dense structure through the consolidation of the impact of subsequent particles on the first layer and particles [18,21].

The surface roughness of coatings can be controlled by altering the coating processing conditions. In order to decrease the surface roughness of coatings, additional polishing is carried out after the processing of the coating. Similarly, for the coating on the inner parts of plasma etching equipment, it is essential to control the surface roughness of the plasma-resistant coating according to the plasma etching processing conditions; these include the reactive gas type, gas flow rate, RF power, etc. This is because the surface roughness of plasma-resistant coatings is considered to be a crucial factor, representing a critical dimension and minimizing the impact of contaminating particles on the process of plasma etching. However, AD processing leads to the deformation of the substrate and coating during deposition due to the enormous physical energy produced when the particles are ejected from the nozzle; this causes problems in processes such as polishing, which is performed to flatten the coating surface. To solve this problem, we selected an interlayer structure (double-layer coating) that plays an important role in controlling the mechanical properties and residual stress [22,23,24,25].

To clarify the effect of an interlayer on the microstructure and mechanical properties of plasma-resistant coatings, a Y_2_O_3_ interlayer that was produced using an electron beam evaporator was coated onto an Al_2_O_3_ substrate before AD deposition. In this study, we aimed to reduce the distortion on the AD coating and substrate during aerosol deposition via the addition of an interlayer before the processing of the AD coating. To understand the influence of the interlayer on the microstructure, mechanical properties, and relaxation of residual stress during aerosol deposition, the microstructure and mechanical characteristics of two kinds of AD coatings, with and without an interlayer, were systematically investigated.

## 2. Materials and Methods

The interlayer was fabricated using an electron beam evaporator (KoMiCo Co., Ltd., Anseong-si, Republic of Korea) with a Y_2_O_3_ source (purity 99.99%, MiCo Co., Ltd., Anseong-si, Republic of Korea). Before the deposition process, the Al_2_O_3_ substrates (20 × 20 mm square and a thickness of 2 mm), sapphire wafers (a diameter of 4 inches and a thickness of 650 μm), and products (a diameter of 650 mm and a thickness of 25 mm) were ultrasonically cleaned and rinsed in acetone, ethanol, and distilled water for 5 min during each step, respectively. The interlayer deposition chamber was evacuated below 5.0 × 10^−6^ Torr (6.6 × 10^−4^ Pa), and the gas was a mixture of high-purity argon (99.999%) at 10 sccm and high-purity (99.999%) oxygen at 40 sccm. The Al_2_O_3_ substrate and sapphire wafer were fixed to the substrate stage using carbon tape. The product was also fixed to the substrate stage using a jig. The substrate stage was regularly rotated at 5 rpm to fabricate a homogeneous coating, and no heat was applied due to the use of a jig to fix the product. The chamber atmosphere temperature was measured at 150 °C, and the deposition rate of the Y_2_O_3_ source was 2.5 Å/s. The interlayer was deposited using an e-beam evaporator to a thickness of approximately 3 μm and 0.5 μm for the TEM specimen. The AD coating and AD coating with an interlayer were deposited onto the Al_2_O_3_ substrates, sapphire wafer, and the product using an aerosol deposition system (KoMiCo Co., Ltd., Anseong-si, Republic of Korea), respectively. A commercially usable Y_2_O_3_ powder (purity 99.99%, MiCo Co., Ltd., Anseong-si, Republic of Korea) was used in the aerosol deposition process. The average particle size (D_50_) of the powder was 1.5 μm after the heat treatment (1100 °C). The aerosol deposition chamber was evacuated below 5.0 × 10^−2^ Torr (6.6 Pa) using a rotary pump and booster pump, and the carrier gas employed was high-purity nitrogen (99.999%) at 50 slm. The powders were moved from the feeder to the nozzle using the carrier gas, and the powders were thereafter accelerated using the nozzle to the substrate at a few hundred meters per second. The distance from the nozzle to the substrate was set to 20 mm, and the powder feeding rate was maintained at 10 g/min. The performance of stacking deposition led to the formation of the AD coating until approximately 10 μm was reached. To investigate the interlayer effect, AD coatings were equally deposited on the bare Al_2_O_3_ substrate and the interlayer, respectively. The structural phase analysis of both powders and all coatings was performed via X-ray diffraction (XRD, Empyrean, Panalytical B.V., Malvern, UK) with Cu Kα1 radiation (λ = 1.5406 Å). The microstructure of the interface between the coatings and the substrates was examined using field emission scanning electron microscopy (FE-SEM, JSM-IT700HR, Jeol Co., Tokyo, Japan). In order to obtain a more detailed microstructural characterization, a transmission electron microscope (TEM) with an energy-dispersive spectrometer (EDS, JEM-ARM200F, JEOL Co., Tokyo, Japan) was utilized. The TEM specimens were prepared using a focused ion beam (Quanta 3D FEG, FEI, Hillsboro, OR, USA). The flatness of the coating and substrate was determined using a coordinate-measuring machine (CMM, Contura, Carl Zeiss, Land Baden-Württemberg, Germany). The hardness and elastic modulus of the coatings were measured using a nanoindenter (NHT3, Anton Paar, Graz, Austria) equipped with a Berkovich diamond indenter tip at room temperature; in order to obtain measurements, the Oliver–Pharr method was employed. At least 15 measurements were carried out to calculate the average values for each sample, except for the minimum and maximum values. The adhesion of the coatings was measured using a scratch tester (MCT3, Anton Paar, Graz, Austria) equipped with a diamond Rockwell-type indenter, which had a radius of 200 μm at a track length of 3 mm; progressive loads from 1 N to 20 N were used, and the critical load values (Lc) were reported.

## 3. Results and Discussion

**Figure 1:**
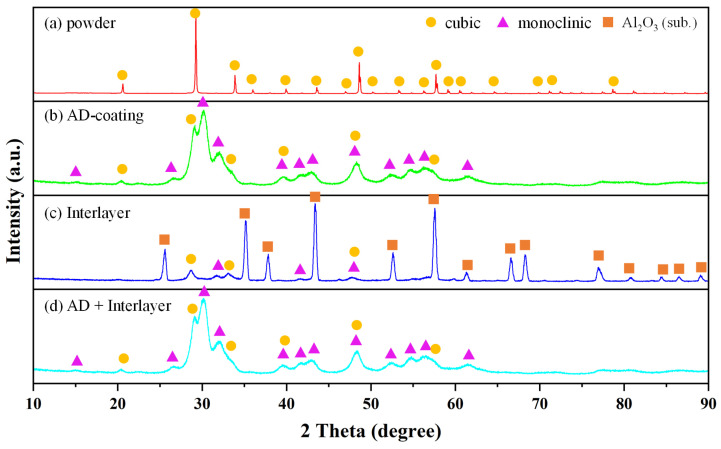
XRD patterns of (**a**) powder, (**b**) AD coating, (**c**) interlayer, and (**d**) AD coating with interlayer.

Figure 1a shows the X-ray diffraction pattern of the raw Y_2_O_3_ powder before aerosol deposition. It only exhibited a Y_2_O_3_ cubic phase due to the powder heat treatment, which was used to increase the high hardness before aerosol deposition [26]. The crystallographic details of the Al_2_O_3_ substrate and the Y_2_O_3_ interlayer grown using the E-beam evaporator are shown in Figure 1c. Structural information regarding the Al_2_O_3_ substrate is included due to the thinness of the interlayer. The X-ray diffraction pattern of the Y_2_O_3_ interlayer showed a mixed cubic and monoclinic phase. Figure 1b,d show the X-ray diffraction patterns of the Y_2_O_3_ coating deposited on the Al_2_O_3_ substrate without and with an interlayer using an aerosol deposition process, respectively. The similar characteristics that can be observed in Figure 1b,d suggest that the interlayer has no impact on the crystalline nature of the Y_2_O_3_ deposition layers. However, the patterns in Figure 1b,d show both cubic and monoclinic phases. Moreover, there are broader peaks in the cubic phase than in the X-ray diffraction pattern of the raw powder; this indicates that the particles were broken and that the grain size in the coating layer was reduced [15]. During aerosol deposition, a hammering effect is created by the particle crushing that occurs and the pressure on the substrate, which causes the phase to change from cubic to monoclinic. Additionally, it has been suggested that a pressure-induced phase shift in Y_2_O_3_, from cubic to monoclinic, occurs at pressures over 13 GPa at room temperature [27].

**Figure 2:**
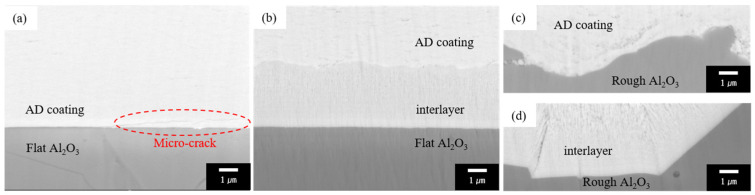
Cross-sectional SEM micrographs of the coating layers after ion milling: (**a**) AD coating, (**b**) AD coating with interlayer, (**c**) AD coating on rough Al_2_O_3_, and (**d**) AD coating with interlayer on rough Al_2_O_3_.

The cross-sectional SEM images of the AD-prepared Y_2_O_3_ layers with and without an interlayer are illustrated in Figure 2a–d. In Figure 2a, it is clear that a microcrack developed during the AD processing (marked by a dotted circle in Figure 2a). It is believed that the formation of microcracks is caused by the internal stress caused by the crushing and cracking of particles during AD processing [28]. The interlayer in Figure 2b clearly shows a columnar structure, which is characteristic of physical vapor deposition [29]. Additionally, no micro horizontal crack around the interface between the Y_2_O_3_ interlayer and the AD coating can be observed. Based on this result, it is suggested that when aerosol particles are pulverized and deposited on a substrate, the pre-deposited Y_2_O_3_ interlayer contributes to the relaxation of stress. Figure 2c,d show the interface regions of the AD-coated Y_2_O_3_ layer and the Y_2_O_3_ interlayer on rough Al_2_O_3_ substrates, respectively. Although both the AD-coated layer and interlayer have the same cubic structure in the Y_2_O_3_ phase, Figure 2c,d show differences in their surface properties; this is a result of the different methods utilized in order to process the coating for the AD coating layer (AD) and the interlayer (E-beam deposition). The Y_2_O_3_ coating layer formed on the surface of the rough substrate via AD coating processing has relatively fine pores. On the other hand, the Y_2_O_3_ interlayer formed via the use of an e-beam evaporator, which represents a physical vapor deposition method, shows a dense structure without pores on the surface of the rough substrate. These differences in microstructural features can affect mechanical properties, such as coating adhesion.

**Figure 3:**
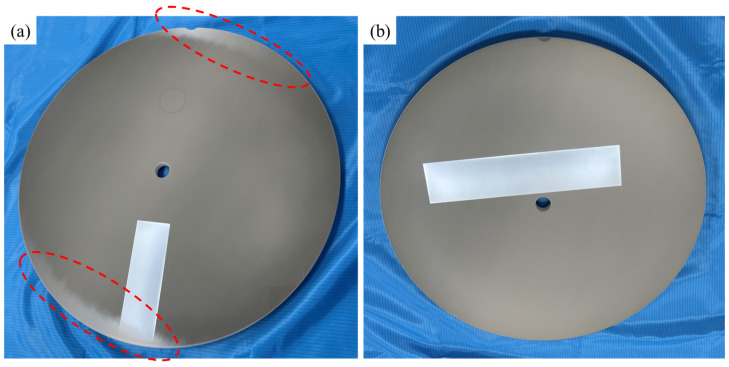
Optical images of products coated with (**a**) AD coating and (**b**) AD coating with interlayer after polishing.

Table 1 details the flatness of the AD coating, interlayer, and AD coating with an interlayer on a 4-inch sapphire wafer; this was measured using a coordinate-measuring machine. Bare sapphire wafers were also measured to identify the variation in the flatness of the AD coating, interlayer, and AD coating with the interlayer. The flatness of the AD coating increased significantly from 12.8 ± 1.5 µm to 228.5 ± 4.4 µm; in addition, it exhibited a convex shape. This significant variation in flatness was caused by the impact energy exerted on the sapphire wafer during aerosol deposition, with a velocity of a few hundred meters per second. On the other hand, the flatness of the interlayer that was created using the physical vapor deposition method slightly increased from 11.2 ± 1.4 µm to 18.2 ± 2.2 µm. In addition, the change in the flatness of the AD coating with an interlayer was less pronounced than that of the AD coating without an interlayer, increasing from 11.6 ± 1.2 µm to 154.6 ± 3.9 µm. The presence of an interlayer significantly enhanced the flatness of the AD coating by approximately one-third. This result can be attributed to the cushioning effect [30], which decreases the kinetic energy of crushed particles during AD processing; this is because the hardness of the interlayer is lower than that of the Al_2_O_3_ substrate. The bent products with convex shapes were adversely affected by the following processes: polishing, which was performed to reduce the roughness of the coating surface. Figure 3a,b show the products formed using the AD coating and AD coating with an interlayer, which is a component of the semiconductor equipment chamber; this is following the polishing procedure that was performed to reduce the roughness of the coated surface. Due to the high flatness and convex shape of the coated product, the surface roughness of the AD coating was uneven when the AD coating was turned upside down and polished; this is highlighted by the dotted circle in Figure 3a. When the product has a convex shape, there will be a height difference between the edge and the center regions. Therefore, when the product is turned over and polished, the center coating surface comes into contact with the polishing pad, but the edge coating surface remains at a distance from the polishing pad; this results in an uneven surface. However, the AD coating with an interlayer is polished uniformly throughout the entire product because there is less variation in the flatness of the interlayer; this is shown in Figure 3b.

**Figure 4:**
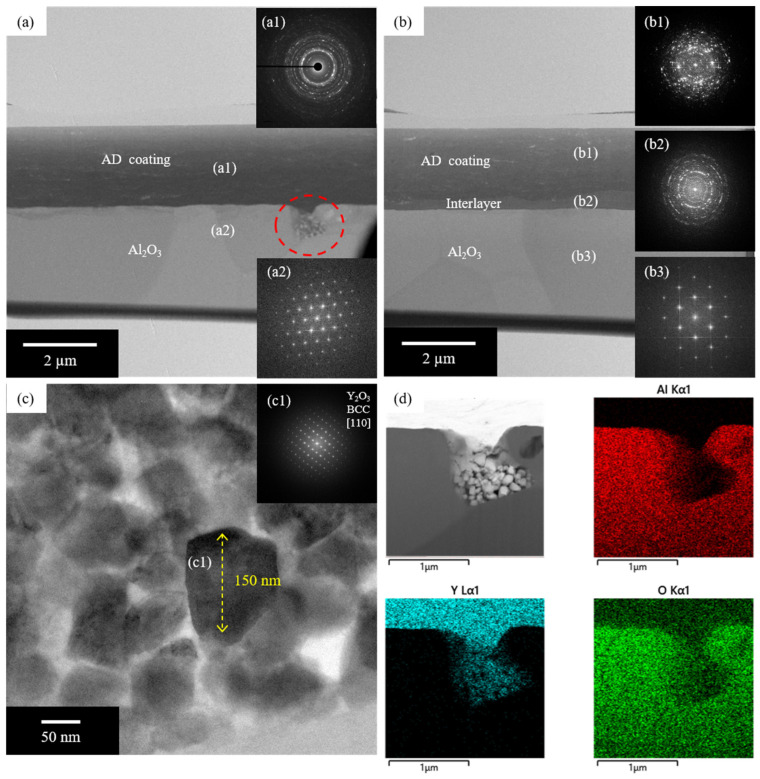
Cross-sectional TEM BF images of (**a**) AD coating and (**b**) AD coating with interlayer with inset (**a1**,**a2**,**b1**–**b3**) SAED patterns; (**c**) high-resolution TEM image of AD coating layer with inset (**c1**) FFT pattern; (**d**) STEM image corresponding to the red-dotted circle region in (**a**) with EDS elemental maps.

Figure 4a,b show cross-sectional TEM bright-field (BF) images of the AD coating and AD coating with interlayer, respectively. The TEM BF image in Figure 4a displays a dark AD coating layer on the bright substrate. The selected area electron diffraction (SAED) patterns obtained from the AD coating layer, which are shown in Figure 4(a1), exhibit the typical diffraction rings of a nanocrystalline, which comprise a mixture of two kinds of Y_2_O_3_ phases with cubic and monoclinic structures. This result coincides with the XRD result shown in Figure 1. The substrate is identified as the Al_2_O_3_ phase, which has a rhombohedral structure; this is shown in the SAED pattern shown in the inset of Figure 4(a2). During AD processing with Y_2_O_3_ particles, a large number of particles are crushed and pulverized by a significant quantity of physical energy; this is because the particles bump into the substrate and themselves. Consequently, the microscale Y_2_O_3_ particles with a cubic structure are deposited onto the nanocrystalline Y_2_O_3_ phase, which has cubic and monoclinic structures, during AD processing. Figure 4b shows the TEM BF image of the Y_2_O_3_ AD coating with a Y_2_O_3_ interlayer that was deposited onto the Al_2_O_3_ substrate. The AD coating layer and interlayer exhibit complex diffraction ring patterns that correspond to a mixture of nanocrystalline Y_2_O_3_ phases with cubic and monoclinic structures, as shown in the SAED patterns visible in Figure 4(b1,b2); these are similar to those of the Y_2_O_3_ phases in the AD coating, as shown in Figure 4a. The substrate in Figure 4b is also identified as the Al_2_O_3_ phase, which has a rhombohedral structure (inset SAED pattern in Figure 4(b3)). Based on this result, it is clear that the addition of an interlayer that has been deposited via e-beam evaporation onto the substrate has no significant influence on the formation of a Y_2_O_3_ AD coating layer. Figure 4c shows a high-resolution TEM image that corresponds to the dotted circle in Figure 4a; this reveals that the accumulated Y_2_O_3_ particles have a length of 100~150 nm. As shown in the inset of Figure 4(c1), the fast Fourier transform that was performed for the particle revealed diffraction spots that exhibited the cubic structure of the Y_2_O_3_ particle. Moreover, the scanning TEM image and corresponding EDS elemental maps shown in Figure 4d indicate that Y_2_O_3_ particles accumulated in the micro-crater on the surface of the Al_2_O_3_ substrate. This result implies that the Y_2_O_3_ particles that accumulated in the crater during AD processing could not grow into a dense coating layer due to insufficient physical energy, which was required to bond the particles together. On the other hand, physical vapor deposition methods such as e-beam evaporation consistently generate a dense interlayer on a rough substrate with craters or dents, as shown in Figure 2d. Therefore, the addition of an interlayer before AD processing via e-beam evaporation enables the craters in the rough substrate to be filled and a good coating layer to be developed.

The hardness and elastic modulus of both the AD coating and the AD coating with an interlayer were evaluated using nano-indentation; these results are summarized in Table 2. The thickness of the coating subjected to nano-indentation was approximately 10 µm. In addition, the maximum depth of the nano-indentation was within 10% of the whole thickness of the coating; this enhanced the accuracy of the measurement [31]. The hardness and elastic modulus of the AD coating were found to be 11.6 ± 0.2 GPa and 208.7 ± 1.6 GPa, respectively. Likewise, the hardness and elastic modulus of the AD coating with an interlayer were found to be 11.7 ± 0.3 GPa and 207.4 ± 1.9 GPa, respectively. The hardness and elastic modulus of the AD coating and the AD coating with an interlayer are similar despite the existence of an interlayer; this is due to the alleviation of the cushioning effect that occurs above the specific thickness of the bottom region. On the other hand, we measured the critical load using a scratch tester to evaluate the adhesion of the coating. The critical load of the AD coating with an interlayer was estimated to be 14.8 ± 0.7 N, which is much higher than that of the AD coating (10.2 ± 0.5 N). These results indicate that the surface hardness and modulus of the AD coating and AD coating with an interlayer are similar to the mechanical properties of the Y_2_O_3_ AD coating layer; however, the adhesion is strongly influenced by the introduction of an interlayer due to the formation of a sound interface between the interlayer and substrate with a dense structure. As a result, the interlayer enhances the adhesion of the total coating layers and substrate.

## 4. Conclusions

In this study, Y_2_O_3_ coatings were deposited via AD processing on a bare Al_2_O_3_ substrate and a substrate with a Y_2_O_3_ interlayer using e-beam evaporation. The crystal structure of the Y_2_O_3_ powders was transformed from cubic to cubic and monoclinic in the Y_2_O_3_ AD coating layer due to the high pressures and physical energies generated during AD processing. Likewise, the Y_2_O_3_ interlayer formed a similar microstructure that consisted of cubic and monoclinic phases. The direct coating of AD on the Al_2_O_3_ substrate generated a micro horizontal crack during the crushing of particles and the fragmentation of powders on the substrate. In addition, the Y_2_O_3_ particles were not densely deposited on the coating layer in the craters of the rough substrate. However, the interlayer deposited via e-beam evaporation formed a dense Y_2_O_3_ coating layer without causing any damage to the substrate. Moreover, the AD coating with an interlayer improved the flatness of the coating and substrate, increasing from 228.5 µm to 154.6 µm. The interlayer did not influence the hardness and elastic modulus but improved the adhesion of the coating, which increased from 10.2 to 14.8 GPa. Based on the present study, it can be concluded that the addition of a Y_2_O_3_ interlayer creates a sound interface between the Al_2_O_3_ substrate and Y_2_O_3_ AD coating layer; this plays an important role in improving the flatness and adhesion of AD coatings, without leading to the degradation of the mechanical properties.

## Figures and Tables

**Table 1:** Flatness of sapphire wafers with AD coating, interlayer coating, and AD coating with interlayer, as measured using CMM.

Coating	Flatness (µm)	
	Before Coating	After Coating
AD coating	12.8 ± 1.5	228.5 ± 4.4
Interlayer	11.2 ± 1.4	18.2 ± 2.2
AD coating with interlayer	11.6 ± 1.2	154.6 ± 3.9

**Table 2:** Hardness, elastic modulus, and critical load of the AD coating and AD coating with interlayer.

Coating	H_IT_ (GPa)	E_IT_ (GPa)	Critical Load (N)
AD coating	11.6 ± 0.2	208.7 ± 1.6	10.2 ± 0.5
AD coating with interlayer	11.7 ± 0.3	207.4 ± 1.9	14.8 ± 0.7

## Data Availability

The original contributions presented in the study are included in the article, further inquiries can be directed to the corresponding authors.
